# Within-Trial Cost-Effectiveness Analysis of a Family-Based Structured Lifestyle Modification Intervention Program for Cardiovascular Risk Reduction: Results from the PROLIFIC Trial

**DOI:** 10.5334/gh.1450

**Published:** 2025-07-31

**Authors:** Ashis Samuel John, Sanjay Ganapathi, Sivadasanpillai Harikrishnan, Thoniparambil Ravindranathanpillai Lekha, Antony Stanley, Biju Soman, Thekkumkara Surendran Anish, Rujuta Hadaye, Jerin Jose Cherian, Nikhil Tandon, Dorairaj Prabhakaran, Panniyammakal Jeemon

**Affiliations:** 1 Achutha Menon Centre for Health Science Studies, Sree Chitra Tirunal Institute for Medical Sciences and Technology, Trivandrum, Kerala, India; 2 Department of Cardiology, Sree Chitra Tirunal Institute for Medical Sciences and Technology, Trivandrum, Kerala, India; 3 Department of Community Medicine, Government Medical College Mananthavady, Wayanad, Kerala, India; 4 Department of Community Medicine, Topiwala National Medical College, Mumbai, India; 5 Division of Developmental Research, Indian Council of Medical Research, New Delhi, India; 6 Department of Global Public Health, Karolinska Institute, Stockholm, Sweden; 7 Department of Endocrinology and Metabolism, All India Institute of Medical Sciences, New Delhi, India; 8 Centre for Chronic Disease Control, New Delhi, India; 9 Public Health Foundation of India, New Delhi, India; 10 Department of Non-Communicable Disease Epidemiology, London School of Hygiene and Tropical Medicine, London, UK

**Keywords:** Ischemic heart disease, Preventive cardiology, Cost-effectiveness analysis, Lifestyle intervention

## Abstract

**Objective:**

We performed a within-trial cost-effectiveness analysis of a targeted family-based structured lifestyle modification intervention for cardiovascular risk reduction.

**Research design and methods:**

The PROLIFIC study was an open-label, cluster randomised controlled trial in the families (first-degree relatives and spouses older than age 18 years) of individuals with premature coronary heart disease. Families in the intervention group received a comprehensive package of interventions facilitated by non-physician health workers: screening for cardiovascular risk factors, structured lifestyle interventions, linkage to a primary healthcare facility for individuals with established chronic disease risk factors or conditions, and active follow-up for adherence. The usual care group received one-time counselling and annual screening for risk factors. The cost was estimated from a health system perspective, including intervention and treatment costs. Effectiveness was measured as changes in risk factors and quality-adjusted life years (QALYs) elicited using the EQ-5D-5 L instrument. The time horizon was two years, and we performed one-way and probabilistic sensitivity analyses.

**Results:**

Over two years, the incremental cost for the intervention compared to usual care was Int$ 157.5 per person (intervention group: Int$ 381.6, usual care group: Int$ 224.1), and the incremental QALY gain was 0.014 (0.0166 Vs 0.0027). The within-trial ICER was 11,352 Int$/QALY. Incremental cost per unit reduction in systolic blood pressure, fasting plasma glucose, HbA1c, total cholesterol, and waist circumference were Int$ 28.5, 26.9, 130.8, 178.7, and 39.8, respectively.

**Conclusions:**

A family-based structured lifestyle modification program yields a net gain in quality of life and is cost-effective at a three times gross domestic product per capita threshold. The intervention is expected to be relatively more cost-effective when scaled up to larger populations over longer time horizons. The intervention has the potential for a substantial public health impact if adopted as a strategy at the state or national level.

Trial Registration Number: Clinicaltrials.gov, NCT02771873.

## Background

Cardiovascular diseases (CVD) are the foremost cause of death and disability in India (1,2). Ischemic heart disease (IHD), a predominant component of CVD, contributes to 110 deaths per 100,000 population in India, which accounts for 16.2% of all deaths (2,3). Furthermore, IHD is responsible for eight percent of disability-adjusted life years (DALYs) lost annually in India (3).

CVDs impose a substantial economic burden worldwide, encompassing both direct healthcare costs and indirect losses from reduced productivity (4,5). In India alone, the cumulative economic loss attributable to CVDs is projected to reach approximately USD 2.17 trillion between 2012 and 2030 (6). A significant proportion of Indian households, nearly one-third, incur catastrophic health expenditures (CHE) when seeking treatment for CVDs (7). The financial toll is particularly severe in the state of Kerala, where up to 92% of lower-income households experience CHE, and as many as 64% resort to distress financing to manage the cost of CVD care (8).

Population-wide risk reduction efforts may help reduce the health system burden from CVD, lower disease-associated out-of-pocket expenditures, and medical impoverishment at the population level (9). A moderate population-level reduction in multiple CVD risk factors such as blood pressure, blood lipids, plasma glucose, and smoking yields a substantial reduction in cardiovascular risk and incidence of CVDs (10,11,12,13). Intervention strategies based on structured lifestyle modification (SLM) are increasingly being evaluated in community settings for cardiovascular risk reduction as they induce healthier choices at the population level (14).

Family-based SLM interventions for CVD risk reduction in high-risk families are promising for multiple reasons (15). Family members of patients with IHD have an increased risk for CVD due to genetics and sharing of environment, behavioural patterns, and belief systems. Lifestyle education delivered to the whole family may yield more impact on behavioural modification than delivery focusing on separate individuals. Lifestyle education also influences the family’s living situation in both structural and environmental aspects. Family-based interventions are, therefore, likely to improve cardiovascular health.

The PROLIFIC trial was an open-label, parallel-group, cluster-randomised controlled trial that evaluated the efficacy of a family-based cardiovascular risk reduction intervention in individuals with a family history of premature IHD in India (16). At the two-year follow-up, the PROLIFIC trial yielded an epidemiologically meaningful increase in the proportion of participants achieving optimal cardiovascular health conditions in the intervention arm relative to the usual care arm. In addition, the intervention group achieved a relatively higher reduction in total cardiovascular risk, as estimated based on the Framingham risk score, compared to the usual care group (17).

In this paper, we present findings from the PROLIFIC study evaluating the within-trial cost-effectiveness and cost-utility of the intervention for cardiovascular risk reduction. Given the substantial potential of the PROLIFIC study intervention to lower the incidence of future cardiovascular events, these findings carry important public health implications. In resource-constrained settings such as low- and middle-income countries, where healthcare budgets are limited, such evidence is essential to inform policy and guide the allocation of preventive care resources.

## Design of the Study

We conducted a within-trial economic evaluation of the PROLIFIC trial. Details of the study, including design, randomisation, follow-up, clinical effectiveness analysis, and economic evaluation plan, have been published elsewhere (16). The study design and reporting comply with the Consolidated Health Economic Evaluation Reporting Standards statement (CHEERS 2022) (18). Ethical clearance for the study was obtained from the Institute Ethics Committee of the Sree Chitra Tirunal Institute for Medical Sciences and Technology, Trivandrum (SCT/IEC/706–2015).

### Study population and setting

Individuals with IHD diagnosed in the preceding year were identified from a tertiary care specialty institute where patients from various parts of Kerala in India seek care. Family members (first-degree relatives and spouses of 18 completed years of age) of individuals diagnosed with IHD before age 55 were randomised into intervention and usual care groups using computer-generated random numbers. Families without at least two members meeting the eligibility criteria and family members who were confined to bed or terminally ill were excluded.

### Intervention and usual care

In the intervention group, trained non-physician health workers (NPHW) visited families assigned to them in bimonthly intervals in the first year and monthly intervals in the following year to deliver the intervention package. The package included screening and detecting cardiovascular risk factors and describing lifestyle options, including diet modification, regular daily exercise, and abstinence from tobacco and alcohol. Recommendations were tailored to the family. The NPHWs helped the families set short-term goals and tracked participants’ progress concerning these goals during follow-up visits.

Other intervention tools used by NPHWs to facilitate intervention delivery were pamphlets, a manual, and a calendar with health messages. Furthermore, the NPHWs used a recipe book designed for the study that provided healthier choices for staple diets appropriate for the region to influence dietary changes. The NPHWs also recorded participants’ blood pressure and random blood glucose levels during home visits, facilitated primary care linkage, and organised 2–3 peer group sessions for individuals with hypertension and diabetes.

Screening for cardiovascular risk factors was done for all participants in the usual care group, and the results were communicated to them. The usual care group participants were also provided a single health education session regarding risk-factor management. Participants with established chronic conditions were linked to the primary healthcare system for treatment and follow-up.

Trained and independent research nurses collected data on demographic and socioeconomic variables, general health status, nutrition and exercise patterns, and substance use using a structured questionnaire. A 5 ml blood sample was collected for laboratory investigations at baseline, one-year, and two-year time points, including a lipid profile, fasting plasma glucose, and glycated haemoglobin levels (HbA1c).

### Study measures

#### Clinical outcomes

All participants’ descriptive health-related quality of life (HRQoL) scores were collected using Euroqol’s EQ-5D-5L questionnaires at baseline, one-year, and two-year time points (19,20). The HRQoL scores were converted to utility values as EQIndex using the EQ-5D-5L value set for India (21). EQIndex was modified by treating values less than zero (worse than death health states) as zero. Quality-adjusted Life Years (QALYs) were estimated as the product of EQIndex and time.

Clinical outcomes included in the cost-effectiveness analysis are the risk factors of IHD (blood pressure, fasting blood sugar, glycated haemoglobin [HbA1c], blood lipids, waist circumference, body mass index (BMI), and Framingham risk score) recorded at baseline, one-year, and two-year time points. The proportion of participants achieving optimal cardiovascular health conditions (meeting or sustaining any three of the following four criteria: blood pressure lower than 140/90 mmHg, fasting blood glucose less than 110 mg/dL, low-density lipoprotein less than 100 g/dL, and abstinence from smoking or tobacco use) were analysed for both the groups.

#### Cost measures

Cost data were collected from a health system perspective. The cost incurred by the public healthcare system catering to the study population and out-of-pocket expenditure borne by participants approaching private healthcare facilities were included in the analysis. The cost of outpatient (OP) consultations, laboratory investigations, medicines, hospital admissions, and procedures were collected separately.

Private healthcare costs borne by the participants were collected directly through baseline and follow-up interviews using structured questionnaires. To estimate expenses incurred by the public health care system, the number of OP visits, laboratory investigations, inpatient (IP) days, and treatments undergone by the patients were multiplied by relevant costs as per the national health system cost database and Ayushman Bharat Pradhan Mantri Jan Arogya Yojana health benefit package rates (22,23).

Intervention costs were collected in a top-down approach under four heads – health worker salary incentives, blood sugar monitoring, sphygmomanometers, and intervention materials – and aggregated to estimate the total cost. The salary incentives covered travel and other incidental expenses incurred by the health workers during intervention delivery.

All the costs were reported in Indian rupees (INR). The values were converted to international dollars (Int$) using purchasing power parity conversion rates for 2017 (24).

### Economic evaluation

For cost-utility analysis (CUA), the incremental cost-effectiveness ratio (ICER) was calculated. The difference in total costs between intervention and usual care groups over two years was divided by the difference in QALYs gained by the two groups to calculate ICER. To deal with missing EQIndex values in the follow-up data, we performed CUA by (i) excluding all cases with missing values (complete case analysis) and (ii) replacing missing values with baseline EQIndex values (replacement analysis). The ICER was calculated from a health system perspective, i.e., only healthcare-related costs were included in the analysis.

To interpret the ICER results, cost-effectiveness thresholds of one-time and three-times India’s per-capita GDP for 2017 were considered. India’s GDP per capita for 2017 is reported as 1.08 lakh INR (Int$6110) by the International Monetary Fund (25). Costs, QALYs, and ICERs are reported using aggregate and per-person values. A discounted cost-utility analysis was done, and a uniform discount of 3% per annum was applied to both costs and QALYs in accordance with Health Technology Assessment in India (HTAIn) reference case (26).

One-way sensitivity (OWSA) and probabilistic sensitivity analyses (PSA) were performed on per-person analysis of complete cases to assess the robustness of CUA. The OWSA was carried out by varying the input parameters by 20% on either side of the base-case value and calculating the corresponding ICER values. A tornado diagram was created by plotting these ICER values against each parameter. The PSA model assigned probability distributions to major input parameters based on the parameter type. We assumed gamma and beta distributions for cost and utility parameters, respectively (Supplementary files Table S1). Using TreeAge Pro software, 10,000 analysis simulations were conducted, and ICER was estimated in each simulation by taking a random parameter value from the assigned distributions. The ICERs thus estimated were plotted on a cost-effectiveness plane with four quadrants (27).

Cost-effectiveness analysis (CEA) was done by dividing the difference in costs between the two treatment groups by differences in change in clinical parameters such as Framingham scores, BMI, systolic and diastolic blood pressure, total cholesterol, low-density lipoprotein (LDL) cholesterol, high-density lipoprotein (HDL) cholesterol, HbA1C, blood glucose, and waist circumference. For the CEA of each parameter, cases with missing values of that parameter in second-year follow-up were excluded.

Microsoft Office Excel 365 (version 2308), R (version 4.2.1), and TreeAge Pro Healthcare 2023 software were used in the analysis.

## Results

Of the 980 patients identified from medical records, 230 were excluded due to lack of documented evidence of IHD (n = 199), heart disease being diagnosed more than one year ago (n = 29), and non-consent (n = 2). The remaining 750 families were randomly assigned to the two treatment groups. There were 825 participants in the intervention (368 families) and 846 in the usual care groups (382 families). The number of participants in the complete case analysis (with available EQIndex values in follow-up 2) was 807 in the intervention and 822 in the usual care groups.

### General characteristics

The mean age of the population was 41 (SD 14) years, and women comprised 66%. The mean years of schooling was 13.3 (SD 3.8) years. Twenty percent of the population were diagnosed with diabetes, and 37% with hypertension. Framingham ten-year risk score of >10% was observed in 27% of the study population.

The mean fasting plasma glucose was 110 (SD 44) mg/dl. Similarly, participants reported a mean HbA1c of 5.93 (1.45) %, systolic blood pressure of 128 (19) mmHg, and diastolic blood pressure of 83 (11) mmHg. The mean BMI was 25.6 (4.5) Kg/m^2^, and the mean Framingham risk score was 12% (14). Mean total cholesterol, LDL cholesterol, and HDL cholesterol were 199 (40), 140 (38), and 47 (12) mg/dl, respectively.

At baseline, the two treatment groups exhibited comparable profiles across key socio-demographic, economic, and clinical characteristics (Supplementary files Table S2).

### Clinical outcomes

The per-person QALYs in the intervention group increased by 0.0166 units in the second year relative to the baseline. In contrast, the usual care group QALY showed a per-person increase of 0.0027 units, yielding a net per-person QALY gain of 0.014 units for the intervention group (Table 1).

**Table 1:** Quality-adjusted Life Years (QALY) gain in the study population.


MEASURE	BASELINE (UC)	FOLLOW-UP (UC)	DIFFERENCE (UC)	BASELINE (IG)	FOLLOW-UP (IG)	DIFFERENCE (IG)	NET GAIN

Aggregate*	738.55	740.79	2.24	715.86	729.26	13.4	**11.157**

Per-person*	0.8984	0.9011	0.0027	0.8871	0.9037	0.0166	**0.014**

**Aggregate** ^#^	759.42	761.66	2.24	732.45	745.84	13.4	**11.157**

**Per-person** ^#^	0.8977	0.9003	0.0026	0.8878	0.9041	0.01623	**0.014**


^*^Complete case analysis (Cases with missing EQIndex values excluded). ^#^Replacement analysis (Missing EQIndex values replaced with baseline values), UC = usual care group, IG = intervention group.

Over the two years of the follow-up, the mean fasting glucose, HbA1c, blood pressure, BMI, and waist circumference increased in the usual care group and decreased in the intervention group. Although the mean total cholesterol decreased in both groups, the magnitude of reduction was higher in the intervention group. Similarly, the Framingham risk score decreased for both groups, with a higher magnitude reduction in the intervention than in the usual care group (Supplementary files Table S3).

The proportion of participants attaining three of four optimal cardiovascular health conditions in the intervention group increased from 50% (411/825) at baseline to 64% at two-year follow-up (514/803). In the control group, the proportion decreased from 47% (399/846) at baseline to 46% (379/819). The net gain in proportion was 15% for the intervention group.

### Costs

The total cost of the intervention was INR 23,46,048 (INT$ 131,704) in the intervention group (INR 2843/INT$ 159.6 per person). The NPHW’s salary incentives and the cost of intervention materials accounted for most (71.9%) of the intervention costs.

The OP costs accounted for 80% of the total treatment cost in the intervention group and 69% in the usual care group. In both years, per-person OP treatment cost was higher in the intervention group, whereas IP cost was higher in the usual care group (Table 2).

**Table 2:** Healthcare and intervention costs in the study population.


COST ITEM	USUAL CARE (AG) (N = 822)	USUAL CARE (PP) (N = 822)	INTERVENTION GROUP (AG) (N = 807)	INTERVENTION GROUP (PP) (N = 807)	INCREMENTAL COST (AG)	INCREMENTAL COST (PP)

**Intervention**						

**Health workers salary**	0	0	11,69,908 (65,673.66)	1,449.70 (81.38)	11,69,908 (65,673.66)	1,449.70 (81.38)

**Blood sugar monitoring**	0	0	78,254.79 (4390.08)	96.97 (5.44)	78,254.79 (4390.08)	96.97 (5.44)

**BP machines**	0	0	1,18,362.7 (6641.61)	146.67 (8.23)	1,18,362.7 (6641.61)	146.67 (8.23)

**Intervention materials: Development**	0	0	4,49,030.9 (25,210.68)	556.42 (31.24)	4,49,030.9 (25,210.68)	556.42 (31.24)

**Intervention materials: Printing**	93,276 (5236.4)	113.48 (6.37)	4,79,309.6 (26,905.38)	593.94 (33.34)	3,86,034 (21,669)	480 (26.97)

**Total cost of intervention**	93,726 (5326)	113.48 (6.37)	22,94,861 (128,831)	2843 (159.6)	22,01,585 (123,594)	2730 (153.3)

**OP Costs**						

**Year 1**	11,20,879 (62,924.78)	1364 (76.57)	11,14,115 (62,545.05)	1381 (77.53)	–6,764 (–379.72)	17 (0.95)

**Year 2**	10,74,432 (60,317.3)	1307 (73.37)	14,33,169 (80,456.35)	1776 (99.7)	3,58,737 (20,139.06)	469 (26.33)

**OP Total**	21,95,311 (123,242)	2671 (150)	25,47,284 (143,001)	3157 (177)	3,51,973 (19,759)	486 (27)

**IP Costs**						

**Year 1**	4,52,000 (25,374.73)	550 (30.88)	2,92,000 (16,392.52)	362 (20.32)	–1,60,000 (–8982.2)	–188 (10.55)

**Year 2**	5,41,000 (30,371.08)	658 (36.94)	3,52,000 (19,760.85)	436 (24.48)	–1,89,000 (–10,610.23)	–222 (12.46)

**IP Total**	9,93,000 (55,746)	1208 (68)	6,44,000 (36,153)	798 (45)	–3,49,000 (–19,592)	–410 (23)

**Total**	32,81,588 (184,224)	3992 (224.1)	54,86,146 (307,986)	6798 (381.6)	22,04,558 (123,761)	2806 (157.5)


Complete case analysis (Cases with missing EQIndex values excluded). Costs expressed in INR (Int$), BP = blood pressure, OP = outpatient, IP = inpatient, Ag = aggregate, Pp = Per–person.

Around 50% of the direct OP treatment costs in both groups were spent on medicines. Consultations and lab investigations accounted for about 20–25% of the direct treatment cost (Supplementary files Table S4).

### Cost-effectiveness

The per-person incremental cost per unit reduction in fasting plasma glucose, HbA1c, total cholesterol, and LDL were INR 480 (Int$ 26.95), INR 2329 (Int$ 130.8), INR 178 (Int$ 10.03), and INR 223 (INT$ 12.54), respectively (Table 3). The incremental cost was INR 708 (Int$ 39.79) and INR 1778 (Int$ 99.86) for a unit reduction in waist circumference and BMI, respectively. The incremental cost for a unit reduction in Framingham risk score was INR 1,26,569 (Int$ 7105) (Table 3). The cost per percentage increase in optimal cardiac health (achievement of three out of four optimal health factors) was INR 1,46,970 (Int$ 8251).

**Table 3:** Incremental cost and cost-effectiveness of the intervention.


VARIABLES	INCREMENTAL COST		INCREMENTAL EFFECTIVENESS		ICER	
		
	AGGREGATE	PER PERSON	AGGREGATE	PER PERSON	AGGREGATE	PER PERSON

**Glucose, mg/dl**	25,56,968 (143,545)	3263.95 (183.23)	–5471	–6.79904	–467.37 (26.24)	–480.06 (26.95)

**HbA1c, %**	25,94,418 (145,647)	3304.85 (185.53)	–1144.16	–1.41869	–2267.53 (127.3)	–2329.51 (130.8)

**Total Cholesterol, mg/dl**	25,56,968 (143,545)	3263.95 (183.23)	–14585	–18.2615	–175.31 (9.84)	–178.73 (10.03)

**LDL, mg/dl**	25,56,968 (143,545)	3263.95 (183.23)	–11,750.2	–14.6113	–217.61 (12.22)	–223.39 (12.54)

**HDL, mg/dl**	25,56,968 (143,545)	3263.95 (183.23)	5767.8	7.163697	443.318 (24.89)	455.62 (25.58)

**Waist Circumference, cm**	22,96,308 (128,912)	2959.93 (166.17)	–3360.67	–4.17584	–683.29 (38.36)	–708.82 (39.79)

**BMI, kg/m** ^2^	22,99,232 (129,076)	2946.96 (165.44)	–1341.75	–1.65666	–1713.6 (–96.2)	–1778.85 (99.86)

**Systolic BP, mmHg**	23,11,108 (129,743)	2943.72 (165.26)	–4701	–5.80374	–491.62 (–27.6)	–507.21 (28.47)

**Diastolic BP, mmHg**	23,11,108 (129,743)	2943.72 (165.26)	–2573.5	–3.17845	–898.04 (–50.41)	–926.15 (51.99)

**Framingham** **Risk Score, %**	25,56,968 (143,545)	3263.95 (183.23)	–20.5908	–0.02579	–1,24,180 (6971.3)	–1,26,569 (7105.4)


Costs expressed in INR(Int$). ICER = incremental cost-effectiveness ratio, HbA1c = glycated haemoglobin, LDL = low-density lipoprotein, HDL = high-density lipoprotein, BMI = body mass index, BP = blood pressure. Positive ICER interpreted as cost per unit increase in the parameter. Negative ICER interpreted as cost per unit decrease in the parameter.

### Cost-utility

In the complete case analysis from a health system perspective, the per-person undiscounted ICER was 2,02,221 INR/QALY (Int$ 11,352). The ICER did not vary (INR 2,04,082 or Int$ 11,457) when missing EQIndex values in the second-year follow-ups were replaced with baseline values (Table 4). The discounted ICERs for the complete case and replacement analysis were INR 2,01,558/QALY (Int$ 11,316) and INR 2,03,460/QALY (Int$ 11,422), respectively. The ICER was lower than three times India’s per-capita GDP (INR 3.26 lakhs, Int$ 18,330) but higher than one GDP per capita (INR 1.08 lakhs, Int$ 6,110).

**Table 4:** Cost-utility analysis.


TYPE OF ANALYSIS	INCREMENTAL COST		INCREMENTAL QALYS		ICER	
		
	AGGREGATE	PER PERSON	AGGREGATE	PER PERSON	AGGREGATE	PER PERSON

**Undiscounted Results**						

Complete Cases*	22,04,558 (123,761)	2806 (157.5)	11.157	0.014	1,97,594 (11,093)	2,02,221 (11,352)

**Replacement Analysis** ^#^	22,05,500 (123,817)	2774 (155.71)	11.157	0.014	1,97,678 (11,097)	2,04,082 (11,457)

**Discounted results (3% discount rate)**						

Complete Cases*	21,99,671 (123,490)	2796 (157)	11.819	0.0139	1,86,118 (10,449)	2,01,558 (11,316)

**Replacement Analysis** ^#^	22,05,500 (123,621)	2765 (155)	11.157	0.014	1,82,512 (10,246)	2,03,460 (11,422)


*Cases with missing EQIndex values excluded. ^#^Missing EQIndex values replaced with baseline values; Costs expressed in INR(Int$); ICER expressed in INR/QALY(Int$/QALY), QALY = quality-adjusted life years, ICER = incremental cost-effectiveness ratio.

Of the total intervention cost (23.46 lakh INR), INR 4.59 lakhs (19.6%) were for developing intervention materials, which did not change with population size. Hence, a scenario analysis was done for different population sizes (1000, 2000, 5000, 7500, 10000) by altering the treatment costs, intervention costs other than development costs, and QALYs. ICER decreased from INR 1.98 lakhs/QALY (Int$ 11,130.54) for a population of 1000 to INR 1.73 lakhs/QALY (Int$ 9,708.28) for a population of 10,000 (Supplementary files Figure S1).

**Figure 1:**
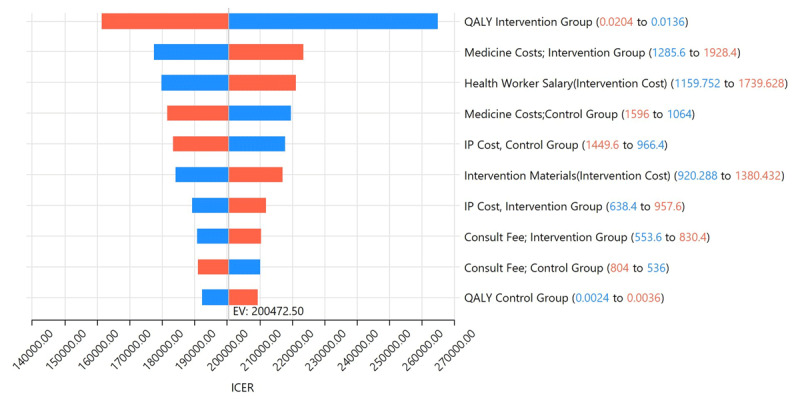
Tornado diagram representing the one-way sensitivity analysis. EV = expected value.

In the one-way sensitivity analysis, input parameters with maximum impact on the estimated value of ICER were QALY generated by the intervention group, the cost of medicines in the intervention group, the cost of health worker salary, and the cost of intervention materials. The ICER decreased with an increase in QALY gain by the intervention group and decreased with an increase in medicine cost in the intervention group and health worker’s salary. The ICER values did not cross the CE threshold of 3 GDP per capita at different input parameter values (Figure 1).

**Figure 2:**
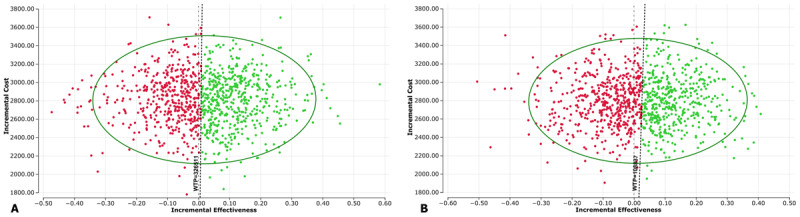
ICE Scatterplot from 1000 probabilistic sensitivity analysis iterations. **(A)** CE threshold 3 GDP per capita. **(B)** CE threshold 1 GDP per capita. WTP = willingness to pay threshold.

The ICE scatterplot of the probabilistic sensitivity analysis showed 55.4% of the simulations in the North-east quadrant, which represents higher effectiveness and higher cost for intervention than usual care, and 44.6% in the North-west quadrant, which means higher cost and lower effectiveness for the intervention. More than half (52.8%) of the simulations were under the CE threshold of 3 GDP per capita for India, and 45.8% were under the CE threshold of 1 GDP per capita (Figures 2A and 2B).

## Discussion

In our within-trial cost-utility analysis of the PROLIFIC trial, family-based SLM interventions for cardiovascular risk reduction yielded a health system ICER of 2,02,221 INR per QALY (Int$ 11,352 per QALY). The PROLIFIC trial interventions are cost-effective at a CE threshold of three times the per capita GDP for India, with the probability of cost-effectiveness as demonstrated by the PSA being 52.8%. Discounting did not alter the interpretations of the ICER estimates. Furthermore, we noticed low to moderate cost-per-unit improvement in cardiovascular risk factors and Framingham risk score in the cost-effectiveness analysis.

Within-trial/model-based CEAs of family-based structured intervention programs for cardiovascular risk reduction were unavailable from LMICs to directly compare our utility/effectiveness improvements and ICER. In a trial-based CEA of the Kerala Diabetes Prevention Program (K-DPP), community-based peer-support lifestyle intervention among individuals at increased risk of type 2 diabetes was cost-effective from the health system perspective at a CE threshold of three times the per capita GDP for India (28). However, the K-DPP interventions did not show a clinically meaningful reduction in the incidence of diabetes (primary outcome) in the overall population. The per-person incremental QALY gain observed in our study (0.014) is lower than the QALY difference (0.04) achieved in the K-DPP trial. The K-DPP interventions cost US$ 50.0 (Int$ 188) per unit gain in QALY from a health system perspective, compared to Int$11,352 in our study. However, the K-DPP study used a Short Form (SF-36) health survey to elicit utility values for QALY estimation compared to EQ-5D-5L in our study. The K-DPP study also used the general area under the curve method to convert SF-36 data into QALYs, while we used India-specific tariffs for EQ-5D-5L data to derive QALYs (28). Another three-year trial of lifestyle interventions to reduce cardiovascular risk documented QALY improvements when estimated using SF-36 (0.07, p = 0.03) but not the EQ-5D-5L tool (0.08, p = 0.24) (29).

The Centre for Cardiometabolic Risk Reduction in South Asia (CARRS) trial evaluated a multicomponent Quality improvement (QI) strategy versus usual care in people with diabetes with poor glycaemic control in India and Pakistan (30). The intervention included improving promptness of treatment modifications by physicians using decision-supported electronic health records (DS-EHR) and enhancing treatment adherence by patients through non-physician care coordinators. The parameters of within-trial economic evaluation included health care system cost (outpatient visits, diagnostic tests, and hospital admissions) and expenses related to the development and delivery of the intervention. The EQ-5D-3L and Health Utilities Index (HUI-3) tools measured health-related quality of life. The undiscounted ICER in the CARRS trial (Int$ 283,038 per QALY gained) was substantially higher than in our study (30).

A stepped wedge trial of a five-month structured cardiovascular risk management program, ‘Healthy Heart’ in the Netherlands, showed a null effect in QALYs gain (31). In the cost-effective analysis of a community-based cardiovascular disease prevention intervention in rural New York and Montana areas with sub-optimal access to medical care, the incremental cost per QALY saved was US$ 62,646 (32). However, this study included only females over 40 with high BMI and a sedentary lifestyle. The intervention was a twice-weekly experiential lifestyle education program emphasizing diet and physical activity behaviours, and the comparator was a lifestyle education program delivered at one-month intervals (32).

In our cost-effectiveness analysis, the cost per unit reduction of systolic blood pressure (SBP) was INR 507.21(Int$ 28.47). Krishnan et al. conducted a retrospective cost-effectiveness analysis of the community-based management of hypertension in Nepal (COBIN study) (33). In the COBIN study, female community health volunteers delivered blood pressure monitoring and lifestyle intervention in the community over one year. The estimated cost per cardiovascular disease DALY averted from a health system perspective was Int$ 1869 when intervention delivery was limited to individuals with hypertension. The cost per DALY averted was Int$ 1321 when intervention was expanded to everyone, irrespective of hypertension status. Further, for both the limited and expanded scenarios, the cost per unit reduction in systolic blood pressure was Int$ 5.41 and Int$ 1.65, respectively (33). In a multi-component intervention study in Argentina, the cost for a 1 mm Hg reduction in SBP was US$ 26 (Int$ 48.57) (34). The CARRS trial reported a cost of Int$ 1223 per additional 5 mmHg SBP reduction, while the K-DPP trial spent Int$ 6.17 per unit reduction of SBP (28,30). The variations in estimates are mainly due to differences in intervention costs (intensity of intervention), utility gains, and measurements employed for assessing utility and costs.

The cost-effectiveness analysis of a diabetes prevention program delivered by community health workers in Africa (Lifestyle Africa) reported a US$ 271 (Int$ 644) per unit reduction in HbA1c (35). In the CARRS trial and K-DPP study, ICERs for a 1% reduction in HbA1c were Int$ 1667 and Int$ 129, respectively. (28,30,36) It was, however, Int$ 2329 for the PROLIFIC study.

In our study, the ICER for 1 mg/dl reduction in total cholesterol and LDL cholesterol was Int$ 10.03 and Int$ 12.54, respectively. For the K-DPP study, costs per unit reduction of total cholesterol and LDL cholesterol were Int$ 753 and Int$ 376, respectively (28,36). In the CARRS trial, lowering LDL cholesterol by 10 mg/dl costs Int$ 1055. (30) The ICER for a unit reduction in waist circumference in the K-DPP study was Int$ 11.24, compared to Int$ 39.79 in our study (28,36).

A major part of the intervention cost of the PROLIFIC study (~20%) was for developing intervention materials, which is a one-time cost that does not change with the number of participants. Scenario analyses keeping this cost component constant demonstrate that the intervention is relatively more cost-effective in a larger population of 10,000 or more.

Although the PROLIFIC study incentivised non-physician health workers, they are part of Kerala’s health care system. Since control of non-communicable diseases is among their primary responsibilities, they can continue providing the intervention in their locality without incurring additional training costs. Thus, the program improved community capacity for cardiovascular risk reduction, but this added value is not incorporated in the current analysis.

The per-person cost for OP treatment was marginally higher in the intervention group compared to the usual care group in the first year. Furthermore, OP treatment cost in the intervention group was substantially higher in the second year. This was probably due to increased health awareness and health-seeking behaviour brought about by the intervention. The IP treatment cost, on the other hand, was substantially lower in the intervention group in both years, and the difference was relatively higher in the second year, pointing toward the long-term impact of the intervention in reducing hospital costs.

While the incremental QALY gain observed at two years (0.014 QALYs) may appear modest, it is important to recognize that improvements in quality of life among apparently healthy or at-risk populations often accrue gradually and may not be fully captured over a short timeframe. Small gains in QALYs at the individual level can translate into meaningful health benefits when sustained over longer periods and across larger populations. From a clinical perspective, this incremental gain reflects improved management of cardiovascular risk factors, which is expected to reduce morbidity and mortality over time. For policymakers, the favourable cost-effectiveness profile combined with even modest quality-of-life improvements justifies investment in scalable lifestyle interventions as a preventive strategy. The long-term cumulative impact on population health and healthcare costs is likely to be substantially greater than the short-term findings. The findings therefore support broader implementation of these programs to address the rising burden of cardiovascular disease.

The significant reduction in CVD risk factors and the estimated 10-year cardiovascular risk, as assessed by the Framingham risk score at the two-year follow-up, highlights the promising long-term potential of the lifestyle intervention model implemented in this study. In the Indian context, where the burden of cardiovascular disease is rising rapidly and access to preventive care is often limited, such programs can serve as a cost-effective and scalable strategy for reducing population-level risk. If the improvements in modifiable risk factors observed over two years are sustained, this approach could substantially lower the future incidence of cardiovascular events, reduce catastrophic health expenditures, and enhance overall health outcomes. Furthermore, embedding these interventions within existing primary care and public health infrastructure may facilitate their long-term integration and sustainability, offering a practical model for noncommunicable disease prevention in low- and middle-income settings.

The study has several limitations. First, the follow-up period of the current study was only two years. Since the overall gain in QALYs over two years for both groups was due to the increase in QALYs in the second year, a longer-term analysis is expected to show a relatively higher magnitude of gain in QALY in the intervention group compared to the usual care group and an increased reduction in in-patient treatment costs. A longer period of follow-up would have detected the sustained effects of positive changes in clinical parameters and Framingham risk scores manifesting as reduced incidence of CVD events in the intervention group, improving the estimated cost-effectiveness of the intervention.

The probabilistic sensitivity analysis revealed that approximately 44.6% of simulations indicated scenarios where the intervention incurred higher costs but yielded lower effectiveness compared to the control. This substantial proportion highlights the uncertainty around the cost-effectiveness estimates and reflects the variability in key parameters such as intervention costs, adherence rates, and risk factor changes. While the base-case results suggest favourable cost-effectiveness, these findings underscore the importance of cautious interpretation, as decision-makers must consider the possibility that the intervention may not always lead to cost savings or improved outcomes. To address this uncertainty, further research with larger sample sizes and longer follow-up periods could help refine these estimates. Additionally, incorporating real-world implementation data may enhance the accuracy and applicability of future economic evaluations. Overall, the PSA results emphasize the need for a balanced approach when interpreting cost-effectiveness findings and reinforce the value of continued monitoring and evaluation during program scale-up.

The risk of contamination common to lifestyle intervention trials, where control group participants may unintentionally be exposed to intervention components through contact with intervention providers, participants, or external sources, is another limitation. This risk is particularly relevant in Kerala, given its high literacy rates and widespread access to print and electronic media. Such contamination would likely bias results toward the null, potentially diluting the observed intervention effects and leading to an underestimation of its true cost-effectiveness.

Since data related to healthcare expenditure each year was collected retrospectively through interviews conducted annually, recall bias may have influenced our overall cost estimation. Interviews conducted at shorter intervals could have minimised this bias. Another limitation is that the study was done from a health-system perspective, and indirect costs such as lost productivity are not included in the analysis.

## Policy Implications

Our results demonstrate that a family-based structured lifestyle modification program is not only effective in improving cardiovascular risk factors and quality of life but also cost-effective within accepted economic thresholds. With an incremental cost of Int$ 157.5 per person and significant gains in QALYs and risk factor reductions, this intervention offers a practical approach to reducing the growing burden of cardiovascular disease in resource-constrained settings. Scaling up such programs at the state or national level in India could lead to substantial public health benefits, including lower incidence of cardiovascular events, reduced healthcare expenditures, and improved population health outcomes. These findings support the integration of family-centred lifestyle interventions into existing health systems as a sustainable strategy for noncommunicable disease prevention.

## Conclusion

In our within-trial economic evaluation, a family-based structured lifestyle modification program delivered by non-physician health workers yields a net gain in quality of life. It is cost-effective at a threshold of three times India’s GDP per capita. The intervention is expected to be relatively more cost-effective when scaled up to larger populations over longer time horizons. The intervention has the potential for substantial public health impact if adopted as a strategy at the state or national level.

## Data Accessibility Statement

Deidentified participant data and the data dictionary will be made available upon a formal request and approval from the Institute Ethics Committee of Sree Chitra Tirunal Institute for Medical Sciences and Technology, Trivandrum. The study protocol is already available in the public domain for free access (https://doi.org/10.1186/s12889-016-3928-6).

## Additional File

The additional file for this article can be found as follows:

10.5334/gh.1450.s1 Supplementary Material. Table S1–S4 and Figure S1.
