# Correction: Within-Trial Cost-Effectiveness Analysis of a Family-Based Structured Lifestyle Modification Intervention Program for Cardiovascular Risk Reduction: Results from the PROLIFIC Trial

**DOI:** 10.5334/gh.1490

**Published:** 2025-10-21

**Authors:** Ashis Samuel John, Sanjay Ganapathi, Sivadasanpillai Harikrishnan, Thoniparambil Ravindranathanpillai Lekha, Antony Stanley, Biju Soman, Thekkumkara Surendran Anish, Rujuta Hadaye, Jerin Jose Cherian, Nikhil Tandon, Dorairaj Prabhakaran, Panniyammakal Jeemon

**Affiliations:** 1 Achutha Menon Centre for Health Science Studies, Sree Chitra Tirunal Institute for Medical Sciences and Technology, Trivandrum, Kerala, India; 2 Department of Cardiology, Sree Chitra Tirunal Institute for Medical Sciences and Technology, Trivandrum, Kerala, India; 3 Department of Community Medicine, Government Medical College Mananthavady, Wayanad, Kerala, India; 4 Department of Community Medicine, Topiwala National Medical College, Mumbai, India; 5 Division of Developmental Research, Indian Council of Medical Research, New Delhi, India; 6 Department of Global Public Health, Karolinska Institute, Stockholm, Sweden; 7 Department of Endocrinology and Metabolism, All India Institute of Medical Sciences, New Delhi, India; 8 Centre for Chronic Disease Control, New Delhi, India; 9 Public Health Foundation of India, New Delhi, India; 10 Department of Non-Communicable Disease Epidemiology, London School of Hygiene and Tropical Medicine, London, United Kingdom

**Keywords:** Ischemic heart disease, Preventive cardiology, Cost-effective analysis, Lifestyle intervention

## Abstract

This article details a correction to: John, A.S., Ganapathi, S., Harikrishnan, S., Lekha, T.R., Stanley, A., Soman, B., Anish, T.S., Hadaye, R., Cherian, J.J., Tandon, N., Prabhakaran, D. and Jeemon, P. (2025) ‘Within-Trial Cost-Effectiveness Analysis of a Family-Based Structured Lifestyle Modification Intervention Program for Cardiovascular Risk Reduction: Results from the PROLIFIC Trial’, *Global Heart*, 20(1), p. 65. Available at: https://doi.org/10.5334/gh.1450.

1. The incremental cost per unit reduction in Framingham risk score was incorrectly reported as INR 1,26,569 (Int$7105). The correct value is INR 1,265.69 (Int$71.05). This affects the Framingham risk score incremental effectiveness and ICER values in Table 3, and changes in data shown in Table S2.
2. The cost per percentage increase in optimal cardiac health, reported as INR 1,46,970 (Int$8,251), is at the group level. The per-person cost is INR 178.15 (Int$10.00).

**Table 3:** Incremental cost and cost-effectiveness of the intervention.


VARIABLES	INCREMENTAL COST		INCREMENTAL EFFECTIVENESS		ICER	
		
	AGGREGATE	PER PERSON	AGGREGATE	PER PERSON	AGGREGATE	PER PERSON

**Glucose, mg/dl**	25,56,968 (143,545)	3263.95 (183.23)	–5471	–6.79904	–467.37 (26.24)	–480.06(26.95)

**HbA1c, %**	–25,94,418(145,647)	3304.85(185.53)	–1144.16	–1.41869	–2267.53(127.3)	–2329.51(130.8)

**Total Cholesterol, mg/dl**	25,56,968 (143,545)	3263.95 (183.23)	–14585	–18.2615	–175.31(9.84)	–178.73(10.03)

**LDL, mg/dl**	25,56,968 (143,545)	3263.95 (183.23)	–11,750.2	–14.6113	–217.61(12.22)	–223.39(12.54)

**HDL, mg/dl**	25,56,968 (143,545)	3263.95 (183.23)	5767.8	7.163697	443.318(24.89)	455.62(25.58)

**Waist Circumference, cm**	22,96,308 (128,912)	2959.93 (166.17)	–3360.67	–4.17584	–683.29(38.36)	–708.82(39.79)

**BMI, kg/m^2^**	22,99,232 (129,076)	2946.96 (165.44)	–1341.75	–1.65666	–1713.6(–96.2)	–1778.85(99.86)

**Systolic BP, mmHg**	23,11,108 (129,743)	2943.72 (165.26)	–4701	–5.80374	–491.62(–27.6)	–507.21(28.47)

**Diastolic BP, mmHg**	23,11,108 (129,743)	2943.72 (165.26)	–2573.5	–3.17845	–898.04(–50.41)	–926.15(51.99)

**Framingham Risk Score, %**	25,56,968 (143,545)	3263.95 (183.23)	–2059.08	–2.579	–1241.80 (69.71)	–1265.69(71.05)


**Table S2:** Changes in Clinical Parameters.


VARIABLES	AGGREGATE CHANGE (UC)	PER-PERSON CHANGE (UC)	AGGREGATE CHANGE (IG)	PER-PERSON CHANGE (IG)

**Fasting Glucose (mg/dL)**	582	0.7106	–4889	–6.0884

**HbA1c (%)**	270.2	0.3303	–874.0	–1.0884

**Total Cholesterol (mg/dL)**	–4043	–4.9365	–18628	–23.198

**LDL (mg/dL)**	887.2	1.0833	–10863	–13.528

**HDL (mg/dL)**	–785.8	–0.9595	4982	6.204

**Waist Circumference (cm)**	530.6	0.6471	–2830.1	–3.529

**BMI (kg/m^2^)**	393.4	0.4785	–948.4	–1.178

**Systolic BP (mmHg)**	952.5	1.1588	–3748.5	–4.645

**Diastolic BP (mmHg)**	465.5	0.5663	–2108	–2.6121

**Framingham Risk Score (%)**	–598.5	–0.7307	–2657.6	–3.309

