# Supplementary material for: Within-Trial Cost-Effectiveness Analysis of a Family-Based Structured Lifestyle Modification Intervention Program for Cardiovascular Risk Reduction: Results from the PROLIFIC Trial
Source: Glob Heart. 2025 Jul 31;20(1):65. doi: 10.5334/gh.1450 (PMC12315683; doi:10.5334/gh.1450)
Supplement: Supplementary Material. — Table S1–S4 and Figure S1. [file gh-20-1-1450-s1.pdf]

Within-Trial Cost-effectiveness Analysis of a Family-Based Structured Lifestyle Modification Intervention Program for Cardiovascular Risk Reduction: Results from the PROLIFIC Trial.

Supplementary Material.

Table S1: Distributions for probabilistic sensitivity analysis

Sl.No	Parameter	Base-case value	Distribution	Alpha	Lambda/Beta
1	Cost of blood sugar monetoring	96.97	Gamma	96.03647	0.990373
2	Cost of BP machines	146	Gamma	96.03647	0.657784
3	Cost of healthworker salaries	1449	Gamma	96.03647	0.066278
4	Cost of intervention materials	1150	Gamma	96.03647	0.08351
5	Cost of intervention in UC	113	Gamma	96.03647	0.846323
6	Consultation fee, UC , Year1	373	Gamma	96.03647	0.25747
7	Consultation fee, UC , Year2	297	Gamma	96.03647	0.323355
8	Consultation fee, IG, Year1	351	Gamma	96.03647	0.273608
9	Consultation fee, IG, Year2	341	Gamma	96.03647	0.281632
10	Lab costs, UC , Year1	273	Gamma	96.03647	0.351782
11	Lab costs, UC , Year2	297	Gamma	96.03647	0.323355
12	Lab costs, IG, Year1	315	Gamma	96.03647	0.304878
13	Lab costs, IG, Year2	370	Gamma	96.03647	0.259558
14	Medicine costs, UC , Year1	657	Gamma	96.03647	0.146174
15	Medicine costs, UC , Year2	673	Gamma	96.03647	0.142699
16	Medicine costs, IG, Year1	627	Gamma	96.03647	0.153168
17	Medicine costs, IG, Year2	980	Gamma	96.03647	0.097996
18	IP Costs, UC , Year1	550	Gamma	96.03647	0.174612
19	IP Costs, IG, Year1	362	Gamma	96.03647	0.265294
20	IP Costs, UC , Year2	658	Gamma	96.03647	0.145952
21	IP Costs, IG, Year2	436	Gamma	96.03647	0.220267
22	Eqindex, UC, Year1	0.898	Beta	15.05169	1.709657
23	Eqindex, IG, Year1	0.887	Beta	15.32318	1.952107

**24**	Eqindex, UC, Year2	0.901	Beta	14.95236	1.642934
**25**	Eqindex, IG, Year2	0.904	Beta	14.84112	1.576048

All costs in INR. UC: Usual Care Group, IG: Intervention Group, BP: Blood pressure, IP: In-patient.

Table S2:Baseline characteristics of the study population

Variables	Usual Care Group(n=846) [mean (SD)]	Intervention Group(n=825) [mean (SD)]
**Age(years)**	41(14)	41(14)
**Males**	292(35%)	268(32%)
**Females**	554(65%)	557(68%)
**Years of schooling**	13.5(3.8)	13.2(3.9)
**Fasting Plasma Glucose(mg/dL)**	112(46)	108(42)
**HbA1c (%)**	5.98(1.5)	5.89(1.39)
**Systolic BP (mmHg)**	128(20)	129(19)
**Diastolic BP (mmHg)**	83(11)	83(11)
**Total Cholesterol(mg/dL)**	199(40)	199(39)
**LDL Cholesterol(mg/dL)**	141(38)	140(37)
**HDL Cholesterol(mg/dL)**	47(12)	48(12)
**Waist Circumference(cm)**	90(12)	89(12)
**BMI (kg/m^2)**	25.5(4.6)	25.7(4.4)
**Framingham Risk Score**	0.12(0.14)	0.11(0.14)

SD= standard deviation; HbA1c=Glycated haemoglobin. BP= Blood Pressure. LDL= low-density lipoprotein. HDL= high-density lipoprotein. BMI= body mass index.

Table S3:Changes in clinical parameters

Variables	Aggregate change (UC)	Per-person change (UC)	Aggregate Change (IG)	Per-person change (IG)
**Fasting Glucose (mg/dL)**	582	0.7106	-4889	-6.0884
**HbA1c (%)**	270.2	0.3303	-874.0	-1.0884
**Total Cholesterol (mg/dL)**	-4043	-4.9365	-18628	-23.198
**LDL (mg/dL)**	887.2	1.0833	-10863	-13.528
**HDL (mg/dL)**	-785.8	-0.9595	4982	6.204
**Waist Circumference (cm)**	530.6	0.6471	-2830.1	-3.529
**BMI (kg/m^2)**	393.4	0.4785	-948.4	-1.178
**Systolic BP (mmHg)**	952.5	1.1588	-3748.5	-4.645
**Diastolic BP (mmHg)**	465.5	0.5663	-2108	-2.6121
**Framingham Risk Score (%)**	-5.98	-0.00731	-26.576	-0.0331

UC= usual care group, IG= intervention group; HbA1c=Glycated haemoglobin. BP= Blood Pressure. LDL= low-density lipoprotein. HDL= high-density lipoprotein, BMI= body mass index.

Table S4: Item-wise OP treatment costs

Year	*Year 1- Ag*	Year 1-Pp	*Year 2-Ag*	Year 2-Pp	Total-Ag	Total- Pp
**Consult Fee (UC)**	3,06,792 (17,223)	373 (20.95)	2,44,278 (13,713)	297 (16.68)	5,51,070 (30,936)	670 (37.64)
**Consult Fee (IG)**	2,83,634 (15,923)	351 (19.73)	2,74,974 (15,436)	341 (19.13)	5,58,608 (31,359)	692 (38.86)
**Lab tests (UC)**	2,24,090 (12,580)	273 (15.30)	2,44,260 (13,712)	297 (16.68)	4,68,350 (26,292)	570 (31.99)
**Lab tests (IG)**	2,54,070 (14,263)	315 (17.67)	2,98,705 (16,769)	370 (20.78)	5,52,775 (31,032)	685 (38.45)
**Medical Exam (UC)**	38,426 (2157)	47 (2.62)	19,795 (1111)	24 (1.35)	58,221 (3268)	71 (3.98)
**Medical Exam (IG)**	45,212 (2538)	56 (3.15)	51,047 (2866)	63 (3.55)	96,259 (5404)	119 (6.70)
**ECG (UC)**	11,290 (5404)	14 (0.77)	12,685 (712)	15 (0.87)	23,975 (1346)	29 (1.64)
**ECG (IG)**	25,540 (1434)	32 (1.78)	17,310 (972)	21 (1.20)	42,850 (2406)	53 (2.98)
**Medicine (UC)**	5,40,282 (30,330)	657 (36.90)	5,53,414 (31,067)	673 (37.80)	10,93,696 (61,398)	1331 (74.69)
**Medicine (IG)**	5,05,659 (28,387)	627 (35.18)	7,91,133 (44,413)	980 (55.03)	12,96,792 (72,799)	1607 (90.21)
**Total (UC)**	11,20,880 (62,924)	1364 (76.55)	10,74,432 (60,316)	1307 (73.38)	21,95,312 (123,240)	2671 (149.93)
**Total (IG)**	11,14,115 (62,544)	1381 (77.50)	14,33,169 (80,455)	1776 (99.70)	25,47,284 (142,999)	3156 (177.20)

All costs in INR(Int$). UC: Usual Care Group, IG: Intervention Group.Ag- Aggregate, Pp-Per-person.

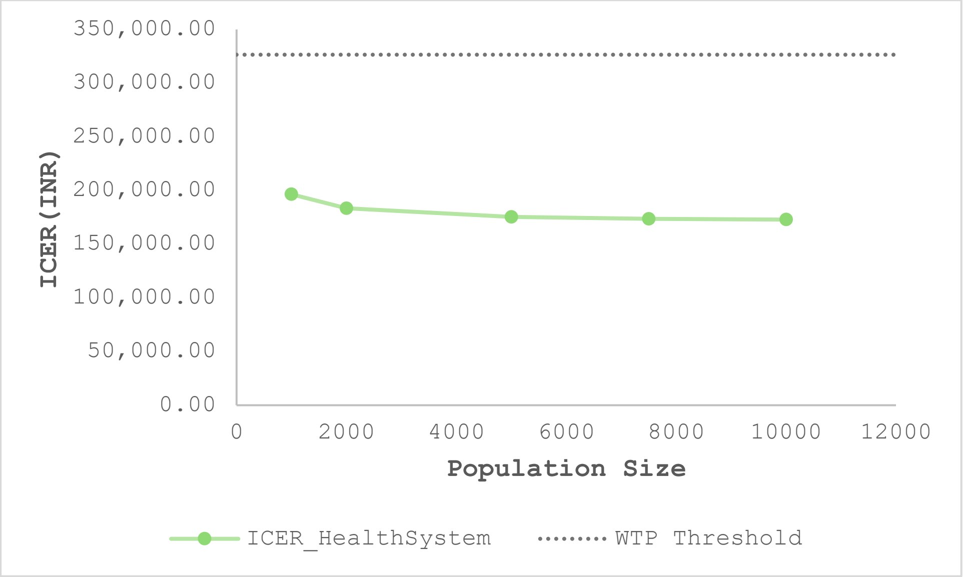

Figure S1:Projected ICERs at various population size
